# First-derivative synchronous spectrofluorimetric method for simultaneous determination of dapagliflozin and sitagliptin in dosage forms and spiked human plasma

**DOI:** 10.1038/s41598-026-61583-7

**Published:** 2026-07-18

**Authors:** Mohamed A. El-Dosoky, Ebrahim A. El-Desouky, Ahmed M. Abdelzaher, Mohamed A. Hasan, Mohamed A. Mahdy

**Affiliations:** Pharmaceutical Analytical Chemistry Department, Faculty of Pharmacy, Al-Azhar University, Nasr City, 11751 Cairo Egypt

**Keywords:** Synchronous spectrofluorimetry, First derivative, Simultaneous determination, Dapagliflozin, Sitagliptin, Spiked human plasma, Chemistry, Drug discovery

## Abstract

The co-administration of Dapagliflozin and Sitagliptin has attracted considerable interest in the management of type 2 diabetes mellitus due to their complementary therapeutic effects. However, their simultaneous determination is analytically challenging because of the significant overlap in their native fluorescence spectra. In this study, a selective and sensitive first-derivative synchronous spectrofluorimetric method was developed for the simultaneous determination of both drugs without prior separation. The proposed approach enabled efficient spectral resolution through zero-crossing points at 348 nm and 289 nm for dapagliflozin and sitagliptin, respectively, using a constant wavelength difference (Δλ = 30 nm). The method exhibited excellent linearity over the concentration ranges of 50–1000 ng/mL and 100–2000 ng/mL for dapagliflozin and sitagliptin, respectively, covering concentration levels relevant to their reported maximum plasma concentrations (Cmax), with low limits of detection (16.02 and 31.07 ng/mL, respectively), indicating high sensitivity. The proposed method demonstrated satisfactory accuracy (mean recoveries of 100.67% and 99.86%) and precision (%RSD < 2%). The method was successfully applied to the analysis of pharmaceutical dosage forms and spiked human plasma, showing reliable recoveries. To the best of our knowledge, this is the first validated spectrofluorimetric method for the simultaneous determination of these co-administered drugs, offering a simple, cost-effective, and efficient alternative to conventional analytical techniques.

## Introduction

Type 2 diabetes mellitus (T2DM) is a chronic metabolic disorder characterized by persistent hyperglycemia resulting from impaired insulin secretion, insulin resistance, or both. It represents a major global health burden with continuously increasing prevalence and significant long-term complications, including cardiovascular disease, nephropathy, neuropathy, and retinopathy^1,2^. According to the International Diabetes Federation, the global prevalence of diabetes is expected to rise dramatically over the coming decades, emphasizing the urgent need for improved therapeutic strategies and effective disease management approaches^3^. Consequently, combination therapy has become a cornerstone in modern diabetic treatment protocols to achieve optimal glycemic control while minimizing adverse effects and delaying disease progression.

Among the currently used antidiabetic agents, dapagliflozin, a sodium-glucose co-transporter 2 (SGLT2) inhibitor, and sitagliptin, a dipeptidyl peptidase-4 (DPP-4) inhibitor, have gained considerable attention due to their complementary mechanisms of action. Dapagliflozin lowers blood glucose levels by inhibiting renal glucose reabsorption in the proximal tubules, thereby increasing urinary glucose excretion, while sitagliptin enhances incretin hormone activity, leading to glucose-dependent insulin secretion and suppression of glucagon release^4,5^. Clinical studies have demonstrated that the co-administration of SGLT2 and DPP-4 inhibitors provides additive glycemic control, improved HbA1c reduction, and better overall therapeutic outcomes compared to monotherapy^6^. This combination is therefore increasingly adopted in the clinical management of T2DM.

From an analytical perspective, the simultaneous determination of dapagliflozin and sitagliptin remains challenging due to their overlapping physicochemical properties and significant spectral interference in their native fluorescence emission profiles. This overlap limits the direct applicability of conventional spectrofluorimetric techniques for their simultaneous quantification without prior separation. Although chromatographic methods such as high-performance liquid chromatography (HPLC) and liquid chromatography–tandem mass spectrometry (LC–MS/MS) have been widely reported for their determination, these techniques are associated with high cost, time-consuming sample preparation, and the need for sophisticated instrumentation, which restrict their routine use in quality control and clinical laboratories^7,8^.

Fluorescence spectroscopy is widely recognized as one of the most sensitive and selective analytical techniques due to its inherent ability to detect trace levels of analytes with minimal sample preparation. Compared with UV–visible spectrophotometry, fluorescence-based methods provide significantly higher sensitivity, lower detection limits, and improved signal-to-noise ratios. In addition, spectrofluorimetric techniques are characterized by simplicity, rapidity, and cost-effectiveness compared to chromatographic and mass spectrometric methods, making them highly suitable for routine pharmaceutical and biological analysis^9,10^. These advantages have driven increasing interest in the development of advanced fluorescence-based analytical strategies for multi-component drug determination.

In this context, synchronous fluorescence spectroscopy (SFS) has emerged as a powerful analytical tool due to its ability to enhance spectral resolution, reduce bandwidth overlap, and improve selectivity in multi-component systems. When combined with derivative processing, first-derivative synchronous fluorescence spectroscopy further enhances peak resolution and minimizes background interference, enabling accurate simultaneous determination of compounds with overlapping emission spectra^11^.

Despite the availability of several analytical methods for the individual determination of dapagliflozin and sitagliptin, there is still a lack of simple, sensitive, and cost-effective spectrofluorimetric approaches capable of simultaneously analyzing both drugs in complex biological matrices. Moreover, most reported methods are not optimized to cover clinically relevant concentration ranges, particularly the maximum plasma concentrations (Cmax), which are essential for evaluating the analytical performance of the proposed method within clinically relevant concentration levels^12^.

To the best of our knowledge, no validated first-derivative synchronous spectrofluorimetric method has been reported for the simultaneous determination of dapagliflozin and sitagliptin. Most of the reported methods rely on chromatographic techniques or conventional spectrophotometric approaches, which may require longer analysis time, more sophisticated instrumentation, or prior separation steps. Therefore, the development of a simple, sensitive, and selective spectrofluorimetric method capable of resolving both analytes without chromatographic separation remains of considerable analytical interest.

**Fig. 1:**
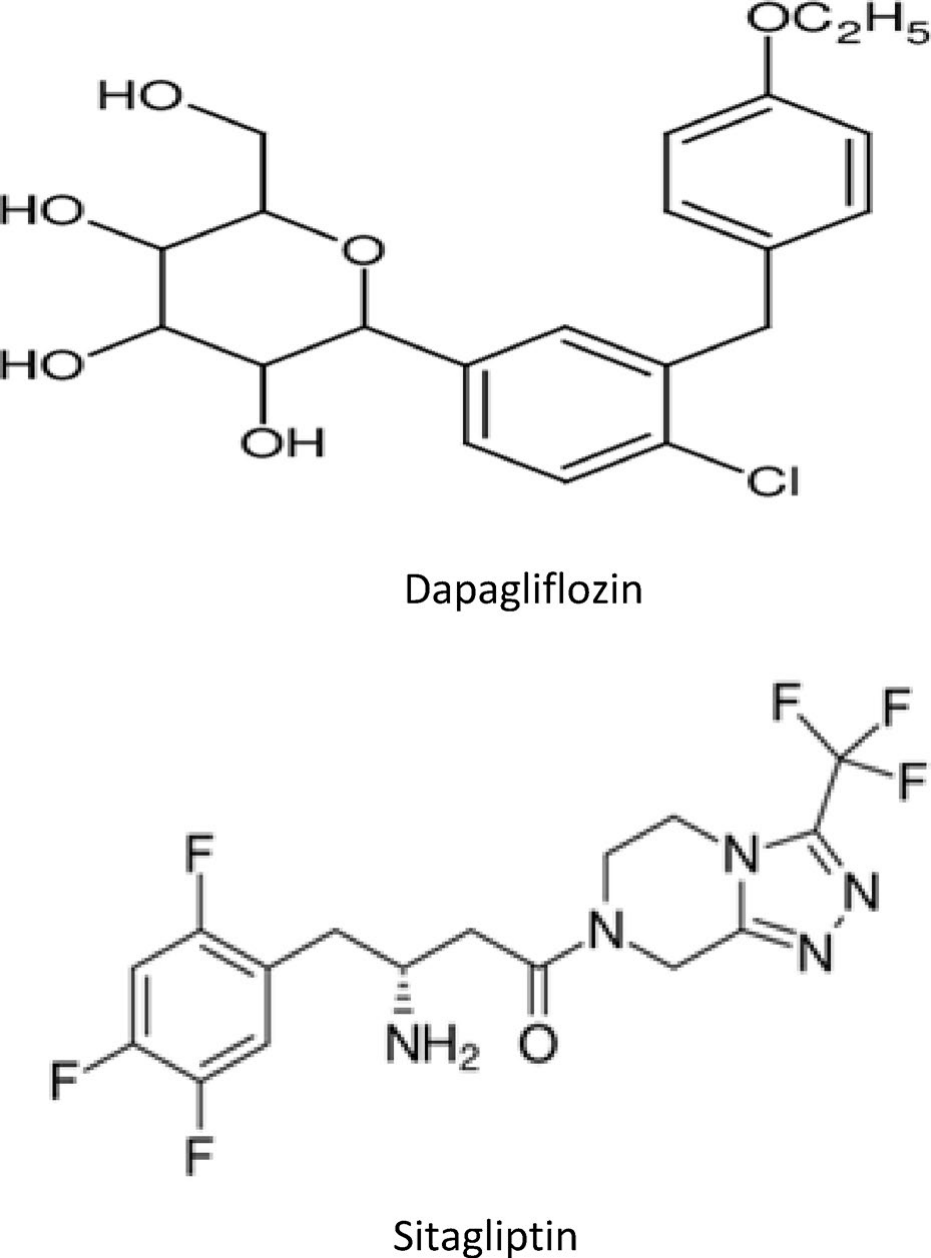
Chemical structures of dapagliflozin and sitagliptin.

Therefore, the present study aims to develop and validate a novel, sensitive, simple and cost-effective first-derivative synchronous spectrofluorimetric method for the simultaneous determination of dapagliflozin and sitagliptin in bulk powder, pharmaceutical formulations, and spiked human plasma without prior separation. The proposed method is specifically optimized to encompass concentration ranges around reported Cmax values of both drugs, ensuring its relevance to clinically encountered concentration levels in plasma samples. In addition, the method adheres to minimize solvent consumption and eliminate the need for expensive chromatographic techniques, while maintaining excellent sensitivity, selectivity, accuracy, and precision. Compared with previously reported analytical methods, the proposed first-derivative synchronous spectrofluorimetric method offers several advantages, including high sensitivity, simplicity, rapid analysis, and low solvent consumption. In addition, the method enables the simultaneous determination of dapagliflozin and sitagliptin without prior separation and was successfully applied to spiked human plasma samples, making it a cost-effective alternative to more sophisticated chromatographic techniques (see Fig. 1).

## Experimental

### Materials

#### Pure samples

Dapagliflozin was kindly provided by EVA Pharmaceuticals (Cairo, Egypt) with a reported purity of 99.80%. Sitagliptin was kindly provided by Andalous Pharma for Pharmaceutical Industries (Cairo, Egypt) with a reported purity of 99.60%.

#### Pharmaceutical preparation

Dapagliflozin-EVA® tablets, labeled to contain 10 mg dapagliflozin per tablet (batch No. 69512), were obtained from the local market. Gliptadalo® tablets, labeled to contain 100 mg sitagliptin per tablet (batch No. 247131), were obtained from the local market.

### Chemicals and reagents

All reagents were of analytical grade, while solvents were of HPLC grade. Freshly distilled water was used throughout the study. Acetonitrile, chloroform, dimethylformamide (DMF), dimethyl sulfoxide (DMSO), ethanol, and methanol (Tedia, USA). Phosphoric acid (Riedel–deHäen, Germany). Glacial acetic acid, boric acid, sodium hydroxide, and hydrochloric acid (El–Nasr Company, Egypt).

#### Preparation of Britton–Robinson buffer

Britton–Robinson (BR) buffer (pH 2–12) was prepared by mixing equal volumes of 0.04 M phosphoric acid, 0.04 M acetic acid, and 0.04 M boric acid. The desired pH was adjusted using 0.2 M sodium hydroxide solution.

### Apparatus

Jasco FP-6200 spectrofluorometer (Japan) equipped with a 150 W xenon lamp. The slit widths for both excitation and emission monochromators were set at 10 nm. Measurements were performed using a 1 cm quartz cell at medium sensitivity. Bench-top centrifuge (TDL-60B, Hunan, China). Rotary evaporator (Scilogex RE100-Pro, USA). pH meter (Jenway 3510, UK) equipped with Ag/AgCl electrode. Analytical balance (Precisa 125 A, Switzerland).

### Standard solution of dapagliflozin and sitagliptin

Stock standard solutions (100 µg/mL) of dapagliflozin and sitagliptin were prepared separately by dissolving 10 mg of each drug in methanol and completing the volume to 100 mL. Working solutions (10 µg/mL) were prepared by appropriate dilution of the stock solutions with methanol.

### Procedures

#### General procedure

Aliquots of dapagliflozin and sitagliptin working solutions (10 µg/mL) were transferred into a series of 10 mL volumetric flasks to obtain final concentration ranges of 50–1000 ng/mL and 100–2000 ng/mL, respectively. To each flask, 2 mL of Britton–Robinson buffer (pH 6) was added, and the volume was completed to mark with methanol. Synchronous fluorescence spectra were recorded using a constant wavelength interval (Δλ) of 30 nm at a scan rate of 400 nm/min. Both excitation and emission slit widths were set at 10 nm. The first derivative of the synchronous fluorescence spectra was obtained, and the peak amplitudes were measured at 348 nm for dapagliflozin and 289 nm for sitagliptin. Calibration curves were constructed by plotting peak amplitude versus drug concentration (ng/mL).

#### Optimization of experimental conditions

The experimental variables affecting fluorescence intensity were optimized by varying one parameter at a time while keeping others constant.

**(i) Selection of optimum Δλ**:

Different Δλ values (10–100 nm) were evaluated using fixed concentrations of dapagliflozin (8 µg) and sitagliptin (15 µg).

**(ii) Effect of solvent**:

Various solvents (acetonitrile, chloroform, DMF, DMSO, ethanol, methanol, and water) were tested under identical conditions.

**(iii) Effect of pH BR buffer**:

The effect of pH was studied using Britton–Robinson buffer in the range of pH 2–12.

**(iv) Effect of BR buffer volume**:

Different volumes of BR buffer (pH 6) were evaluated.

#### Validation of the method

The method was validated according to ICH guidelines^13^.

##### Linearity (Construction of the calibration graph)

Calibration curves were constructed by plotting derivative amplitude versus concentration.

##### Limits of detection and quantitation(LOD and LOQ)

Calculated using:

$$\:\mathrm{L}\mathrm{O}\mathrm{D}\hspace{0.17em}=\hspace{0.17em}3.3\:\:\sigma/\:\mathrm{S}$$

$$\:\mathrm{L}\mathrm{O}\mathrm{Q}\hspace{0.17em}=\hspace{0.17em}10\:\:\sigma/\:\mathrm{S}$$

##### Accuracy and precision

Evaluated using three concentration levels with triplicate measurements.

##### Specificity

Assessed using synthetic mixtures and standard addition technique.

##### Robustness

Evaluated by small variations in Δλ (± 1 nm), pH (± 0.1), and buffer volume (± 0.1 mL).

#### Analysis of pharmaceutical dosage form

Ten tablets were accurately weighed and finely powdered. An amount equivalent to 10 mg of the drug was transferred to a 100 mL volumetric flask, extracted with methanol, filtered, and diluted to obtain a working solution of 10 µg/mL. Aliquots within the linearity range were analyzed using the general procedure, and drug content was calculated using the regression equation.

#### The reported method

Two previously reported UV spectrophotometric methods were used for comparison. Dapagliflozin was determined using ethanol: phosphate buffer (pH 7.2) (1:1, v/v) at 233.65 nm^14^. Sitagliptin was determined using water as solvent at 257 nm^15^.

#### Spiked human plasma procedure

Aliquots (1 mL) of working standard solutions of dapagliflozin and sitagliptin were transferred into 10 mL centrifuge tubes containing 1 mL of drug-free human plasma. Protein precipitation was carried out by adding 3 mL acetonitrile, followed by vortex mixing. The mixture was centrifuged at 4000 rpm for 30 min. The clear supernatant was separated and evaporated to dryness under reduced pressure at 40 °C using a rotary evaporator. The evaporation step was performed to preconcentrate the analytes and improve the sensitivity of the proposed spectrofluorimetric method. The residue was reconstituted in 10 mL methanol. Appropriate aliquots were analyzed according to the general procedure.

Drug-free human plasma was obtained from a certified commercial supplier and used for spiking experiments. No human subjects were involved in this study. Therefore, ethical approval and informed consent were not required. All experimental procedures were performed in accordance with relevant guidelines and regulations.

## Results & discussions

### Spectral characteristics

**Fig. 2:**
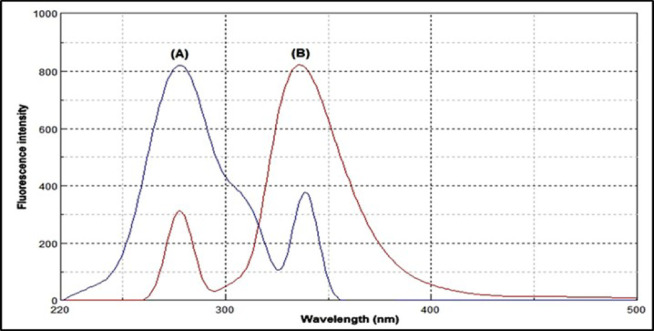
Excitation and emission spectra of dapagliflozin (800 ng/mL) in methanol–Britton–Robinson buffer (pH 6).

**Fig. 3:**
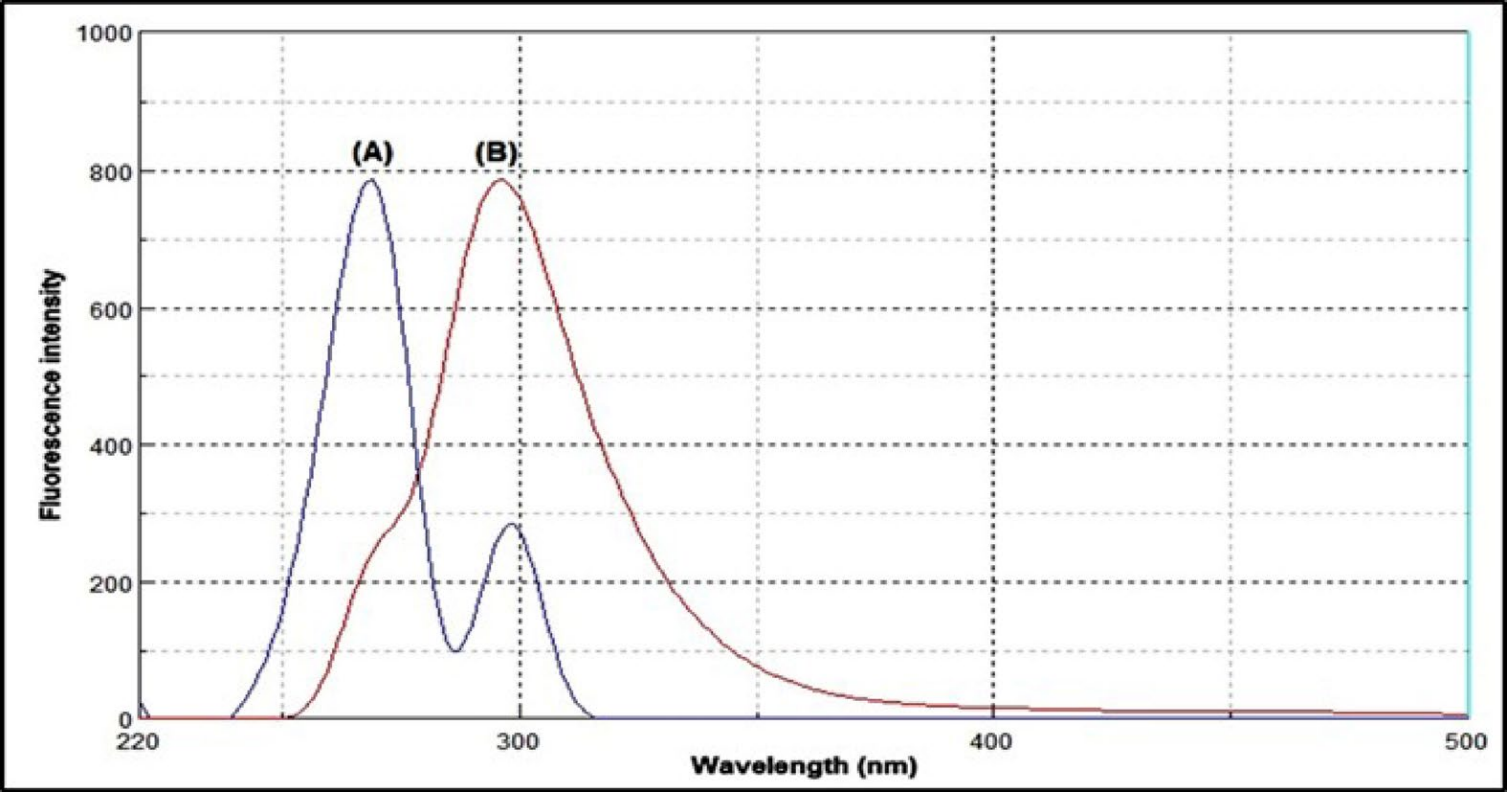
Excitation and emission spectra of sitagliptin (1500 ng/mL) in methanol–Britton–Robinson buffer (pH 6).

Dapagliflozin and sitagliptin exhibit native fluorescence; however, their simultaneous determination using conventional fluorescence spectroscopy is hindered by significant spectral overlap. Dapagliflozin showed an emission maximum at 337 nm upon excitation at 278 nm, whereas sitagliptin exhibited an emission maximum at 298 nm upon excitation at 269 nm (see Fig. 2 and Fig. 3).

**Fig. 4:**
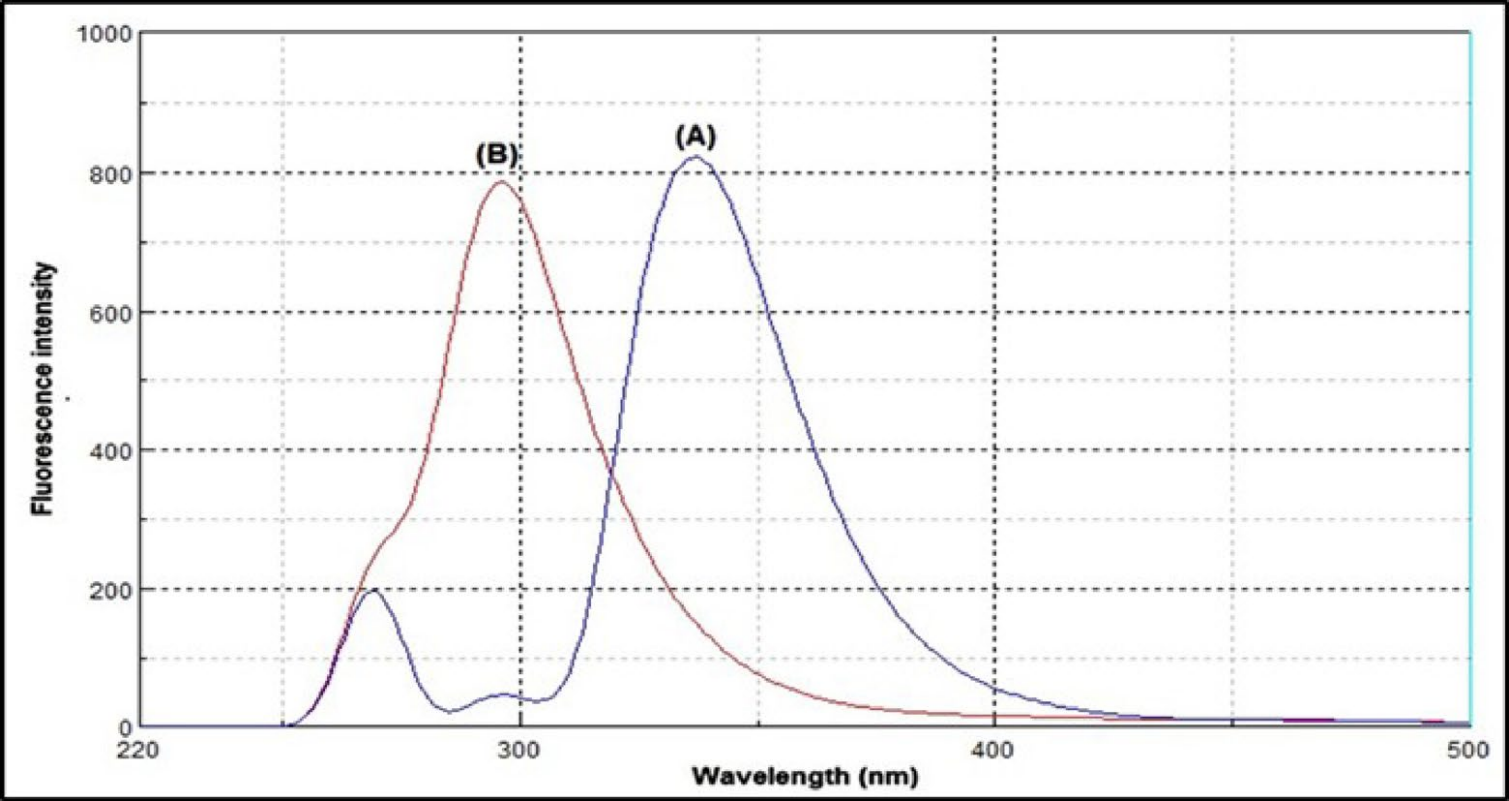
Overlaid emission spectra of dapagliflozin (800 ng/mL) and sitagliptin (1500 ng/mL) showing severe spectral overlap.

As illustrated in Fig. 4, a significant overlap between the emission spectra of both drugs was observed, which prevents their simultaneous determination using conventional fluorescence techniques.

**Fig. 5:**
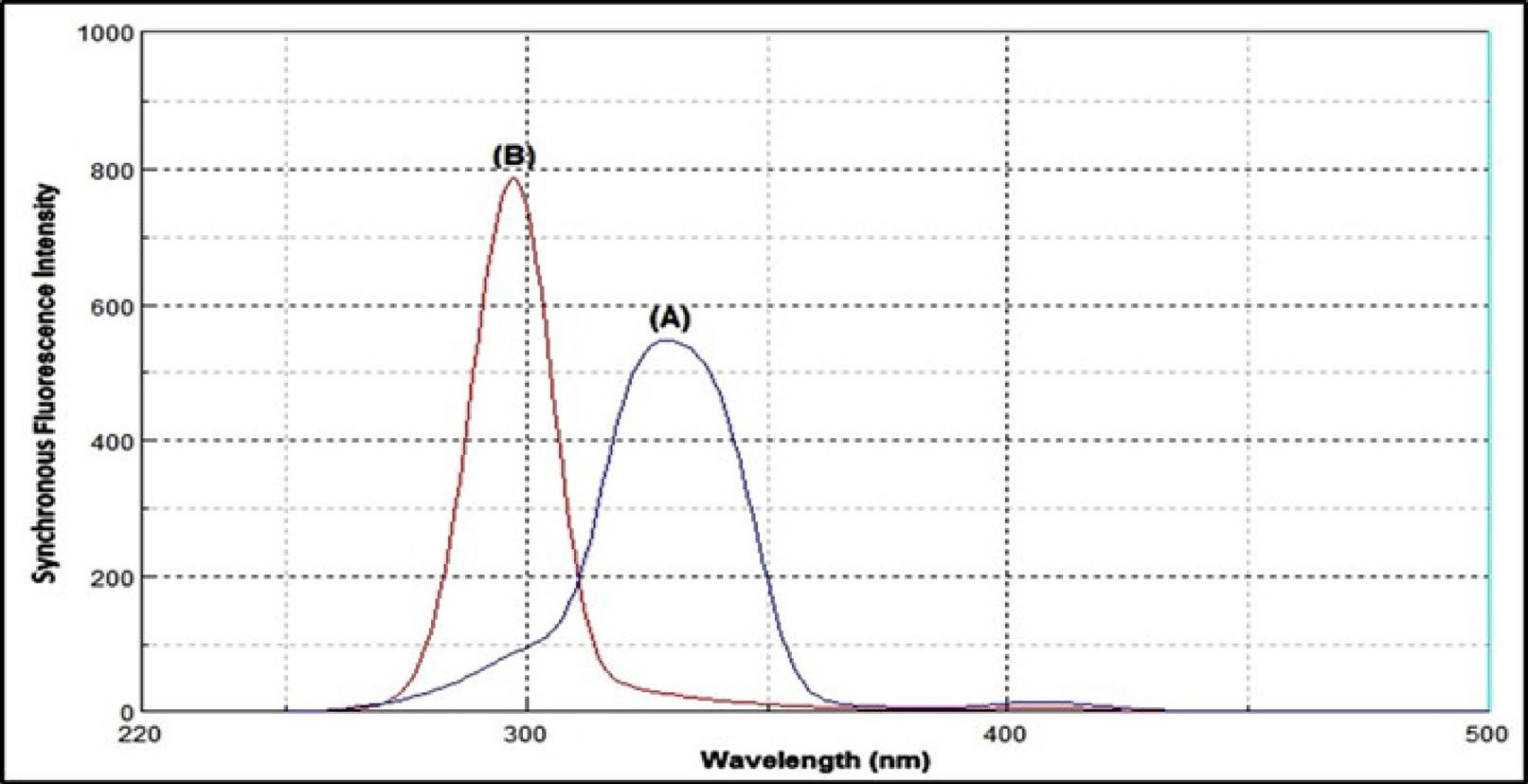
Synchronous fluorescence spectra of dapagliflozin and sitagliptin at Δλ = 30 nm showing partial spectral overlap.

To overcome this limitation, synchronous fluorescence spectroscopy was applied. Although partial improvement in spectral resolution was achieved, considerable overlap still persisted (see Fig. 5).

**Fig. 6:**
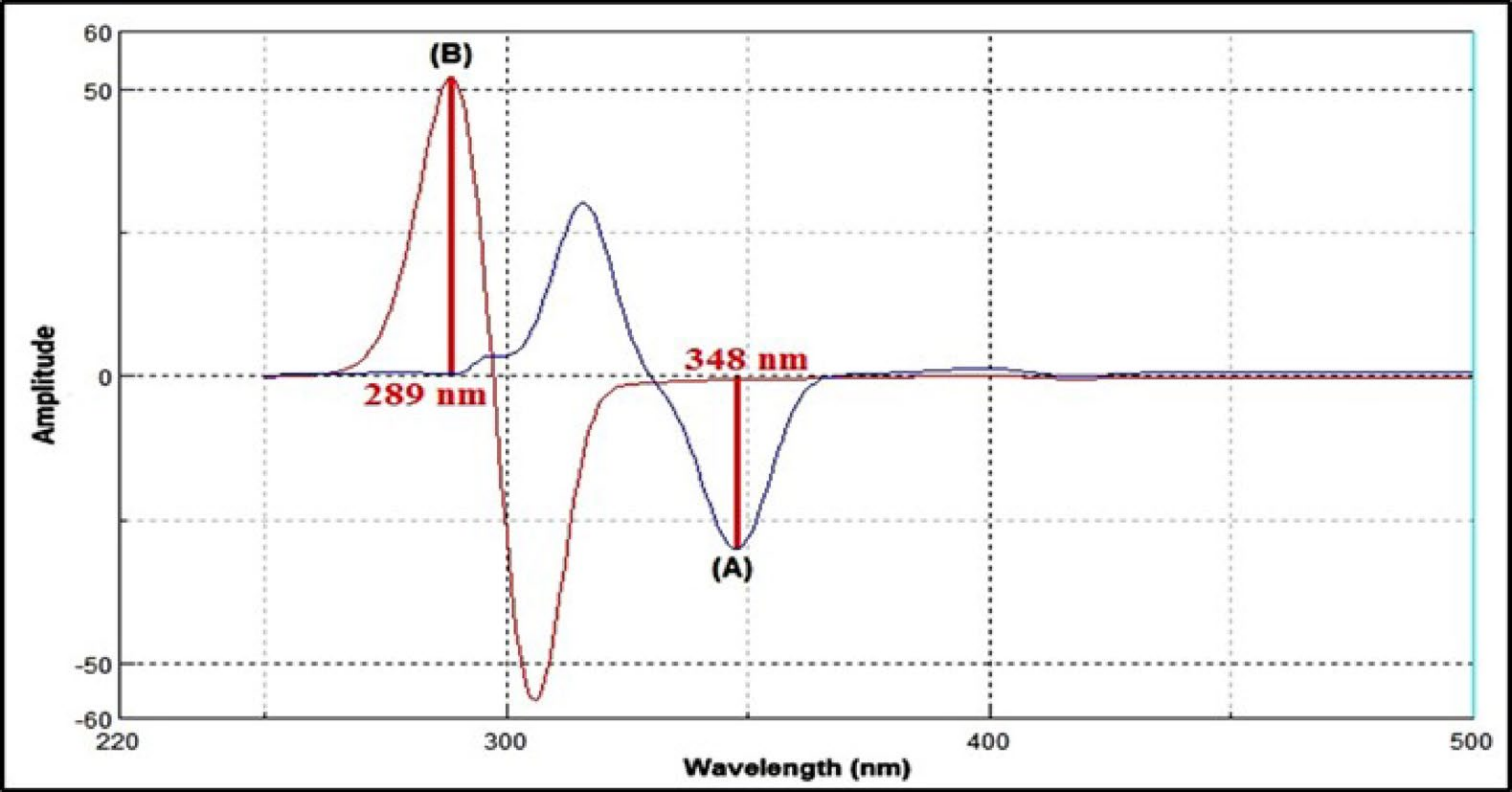
First derivative synchronous fluorescence spectra of dapagliflozin and sitagliptin showing complete spectral resolution with zero-crossing points at 348 nm and 289 nm, respectively.

Further enhancement was achieved by applying first-derivative processing to the synchronous spectra, which resulted in complete resolution of both drugs with well-defined zero-crossing points (see Fig. 6).

This approach enabled the selective determination of dapagliflozin at 348 nm and sitagliptin at 289 nm without mutual interference.

### Optimization of experimental conditions

The experimental variables affecting the fluorescence response were systematically optimized to achieve maximum sensitivity and selectivity.

#### Effect of Δλ

The choice of Δλ plays a crucial role in synchronous fluorescence spectroscopy, as it directly influences spectral resolution and sensitivity. Various Δλ values (10–100 nm) were investigated. A Δλ of 30 nm provided optimal resolution with well-defined peaks and minimal spectral interference, and was therefore selected for further analysis.

#### Effect of solvent

**Fig. 7:**
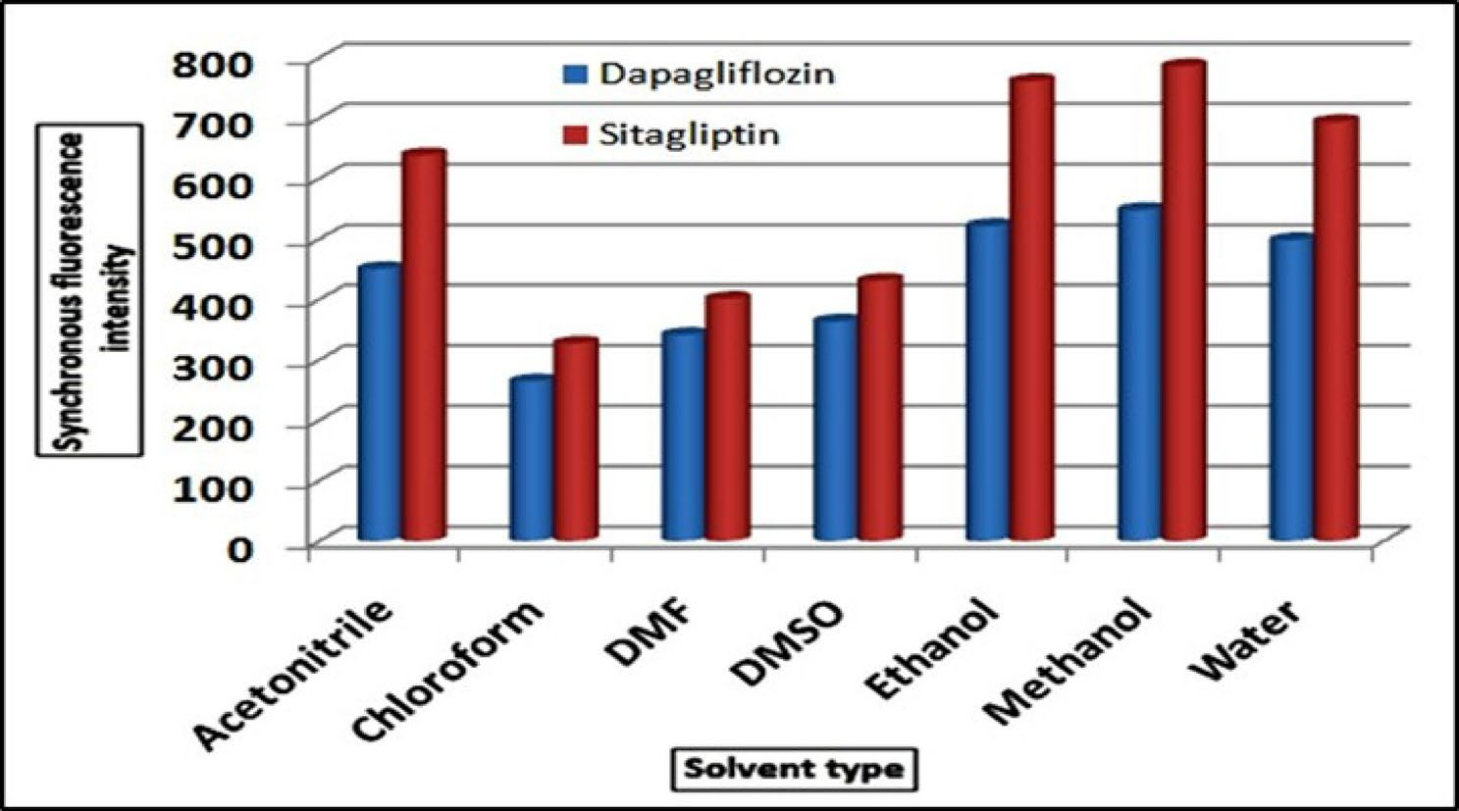
Effect of solvent type on the synchronous fluorescence intensity of dapagliflozin and sitagliptin.

The influence of different solvents on fluorescence intensity was evaluated. Methanol provided the highest fluorescence intensity and best spectral stability for both drugs, which can be attributed to its suitable polarity and its ability to reduce non-radiative decay processes. Although water is considered a greener solvent, methanol provided higher fluorescence intensity and better spectral resolution for both drugs. Therefore, methanol was selected to achieve maximum sensitivity and analytical performance (see Fig. 7).

#### Effect of pH

**Fig. 8:**
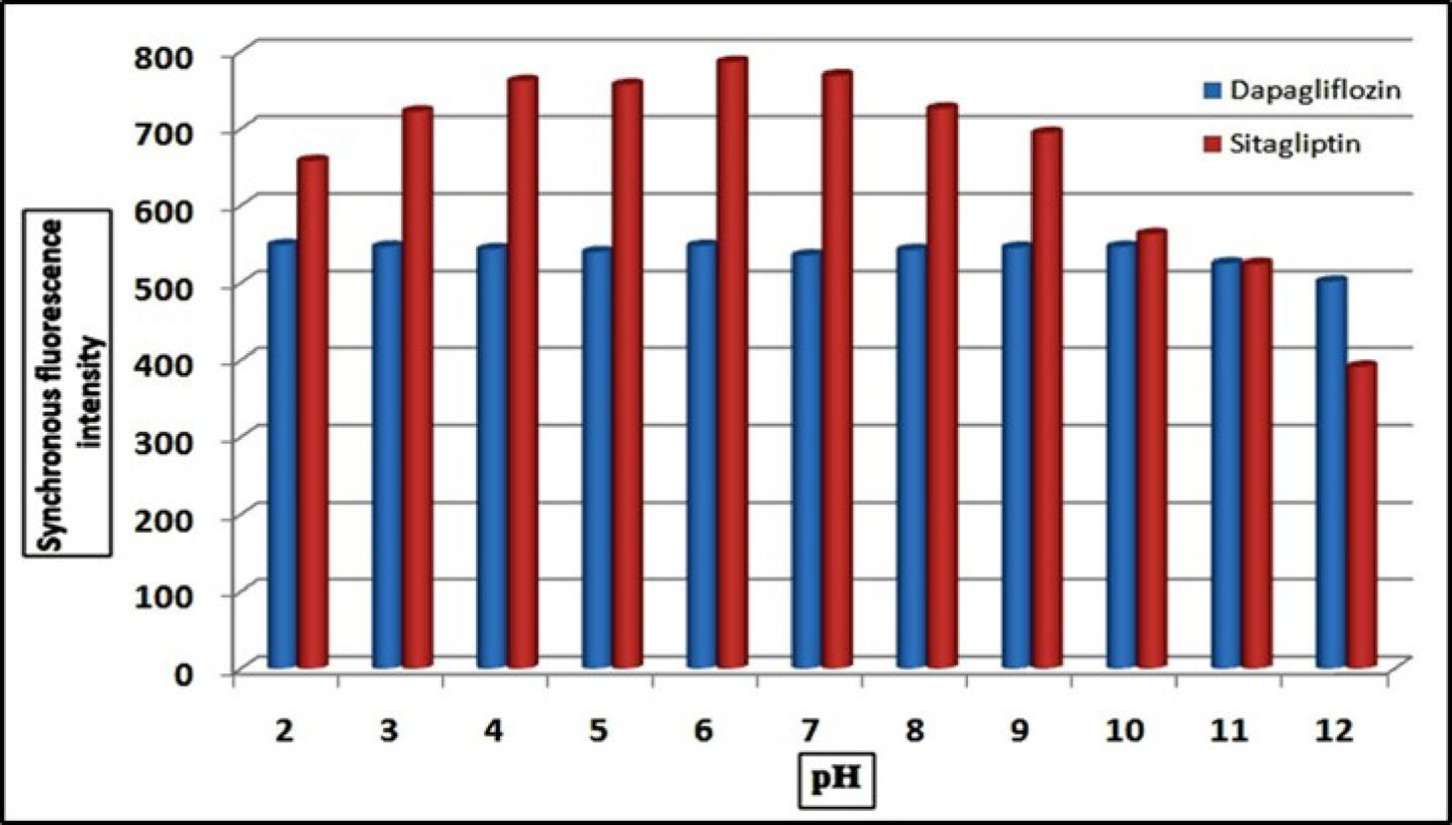
Effect of pH on the synchronous fluorescence intensity of dapagliflozin and sitagliptin.

The effect of pH on fluorescence intensity was studied using Britton–Robinson buffer over the pH range of 2–12. Maximum fluorescence intensity for both drugs was experimentally observed at pH 6; therefore, this pH was selected for all subsequent measurements. Britton–Robinson buffer was selected because it provides a broad and well-controlled pH range, allowing a systematic evaluation of the effect of pH on the fluorescence behavior of both drugs (see Fig. 8).

####  Effect of buffer volume

**Fig. 9:**
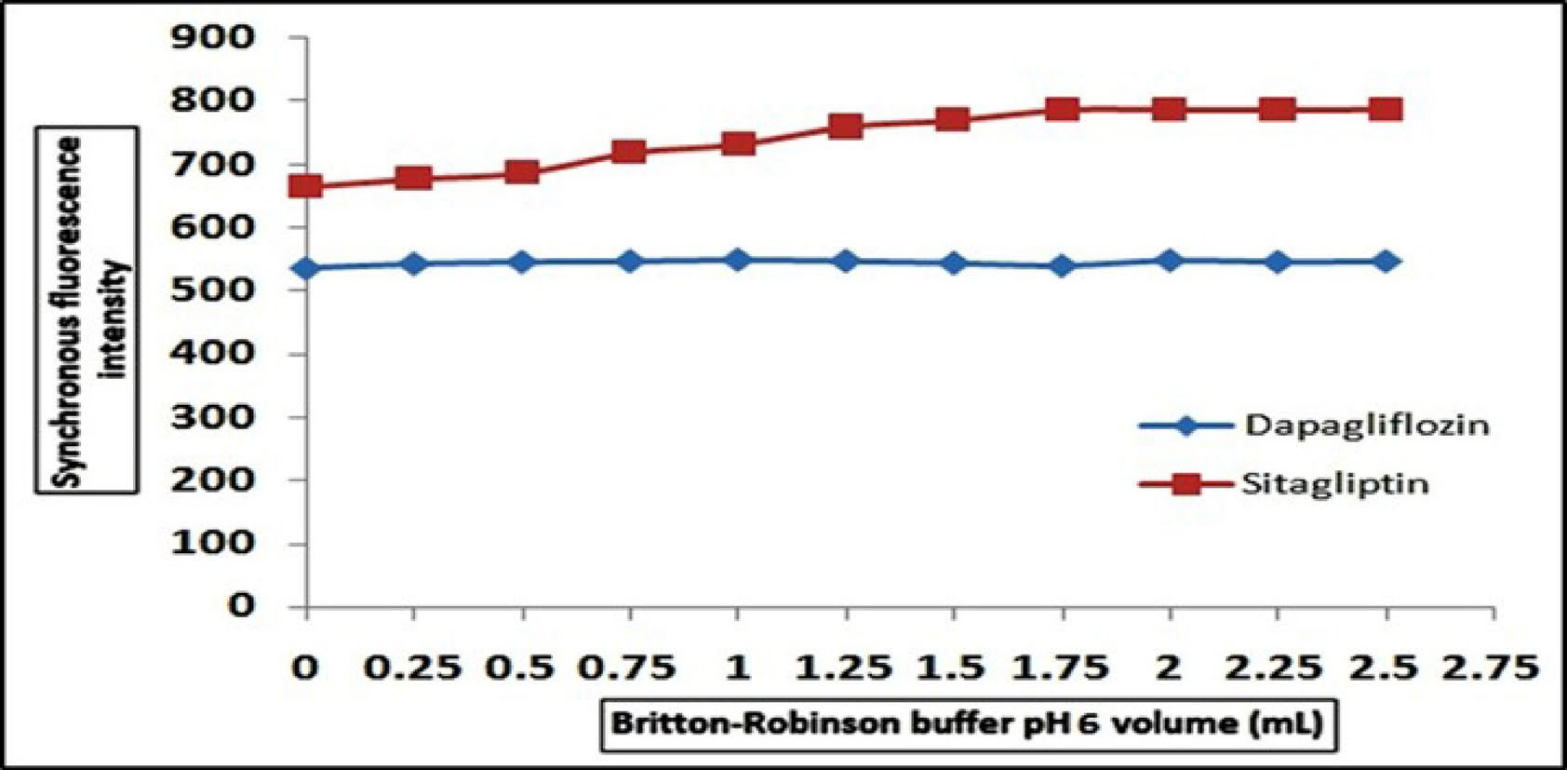
Effect of buffer volume on the synchronous fluorescence intensity of dapagliflozin and sitagliptin.

Different volumes of Britton–Robinson buffer were investigated. A volume of 2 mL was found to be optimal, providing maximum and stable fluorescence intensity (see Fig. 9).

###  Method validation

The proposed method was validated according to ICH guidelines.

#### Linearity and range

**Fig. 10:**
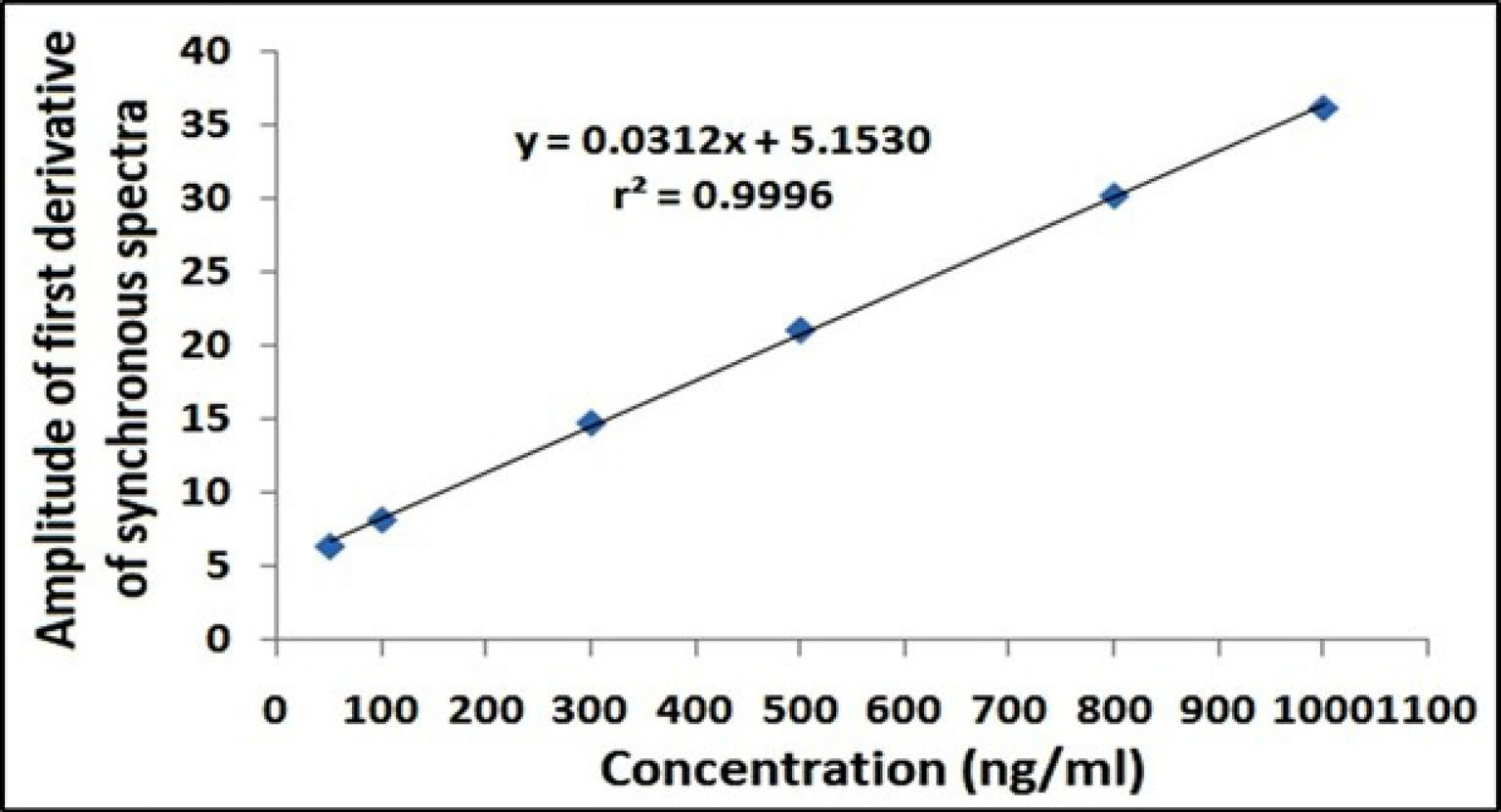
Calibration curve for dapagliflozin using the proposed method.

**Fig. 11:**
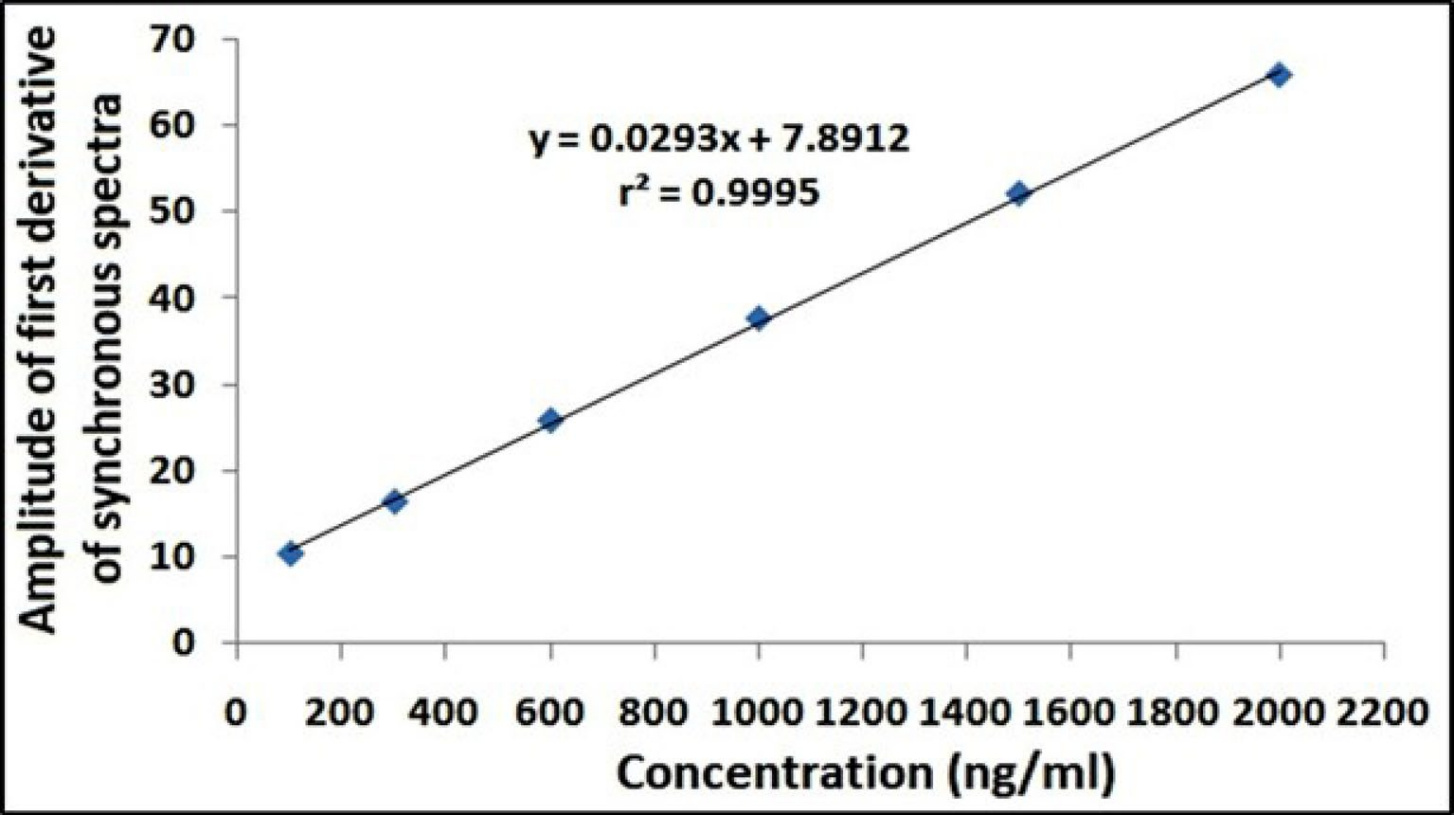
Calibration curve for sitagliptin using the proposed method.

The method exhibited excellent linearity over the concentration ranges of 50–1000 ng/mL for dapagliflozin and 100–2000 ng/mL for sitagliptin, which were selected to cover their reported Cmax values in human plasma. Calibration curves were constructed by plotting the first derivative amplitude against concentration (see Fig. 10 and Fig. 11).

The high correlation coefficients confirm the excellent linear relationship between concentration and response.

#### Detection and quantitation limits (LOD and LOQ)

The low LOD and LOQ values indicate the high sensitivity of the proposed method, making it suitable for trace level analysis in biological samples.

#### Accuracy and precision

The high recovery values and low %RSD demonstrate that the method is both accurate and precise.

#### Robustness

The method remained unaffected by small variations in experimental conditions, confirming its robustness and reliability.

The validation results are summarized in Table 1.

**Table 1:** Regression and validation data for the determination of dapagliflozin and sitagliptin by the proposed first derivative synchronous spectrofluorimetric procedure:.

Parameters	Proposed method	
	Dapagliflozin	Sitagliptin
Wavelength (nm)	348	289
Linearity range (ng/mL)	50 ─ 1000	100 ─ 2000
LOQ (ng/mL)	48.547	94.137
LOD (ng/mL)	16.020	31.065
**Regression Equation**	*y* ^a^ = *b x* ^b^ + *a*	*y* ^a^ = *b x* ^b^ + *a*
**-** Slope (*b*)	0.031	0.029
**-** Intercept (*a*)	5.153	7.891
Coefficient of determination (r^2^)	0.9996	0.9995
Accuracy (% R)^c^	100.67	99.86
**Precision**^d^ **(% RSD)**		
- Repeatability	1.251	1.624
- Intermediate precision	0.742	0.995
**Robustness (% R**^e^ ±**% RSD)**		
- Δλ (± 1 nm)	100.41 ± 1.261	101.34 ± 1.892
- pH (± 0.1)	99.36 ± 1.051	100.55 ± 1.425
**-** Buffer volume (± 0.1 mL)	99.91 ± 0.962	99.41 ± 0.147

^a^ The peak amplitude of the first derivative of synchronous fluorescence spectra.

^b^ Concentration of the drug in ng/mL.

^c^ Average of nine determinations (three concentrations repeated three times).

^d^ %RSD of nine determinations (three concentrations repeated three times).

^e^ Average of three determinations.

#### Specificity

The proposed method demonstrated high specificity, as it was capable of selectively determining each drug in the presence of the other without interference. The application of the standard addition technique confirmed the absence of matrix interference from pharmaceutical excipients.

The obtained results are presented in Table 2.

**Table 2:** Determination of dapagliflozin and sitagliptin in their laboratory prepared mixtures by the proposed first derivative synchronous spectrofluorimetric procedure:.

Dapagliflozin			Sitagliptin		
Added (ng/mL)	Found (ng/mL)	%Recovery	Added (ng/mL)	Found (ng/mL)	%Recovery
50	50.23	100.46	500	492.15	98.43
100	101.61	101.61	1000	987.33	98.73
200	198.12	99.06	200	196.41	98.21
500	491.73	98.346	300	298.09	99.36
600	588.35	98.06	400	394.64	98.66
800	794.12	99.27	100	100.09	100.09
Mean ± %RSD		99.47 ± 1.351	Mean ± %RSD		98.91 ± 0.702

The results of the standard addition technique are shown in Table 3.

**Table 3:** Recovery study of dapagliflozin and sitagliptin by standard addition technique using the proposed method:.

Drug	Pharmaceutical taken (ng/mL)	Pharmaceutical found^a^ (ng/mL)	Pure added (ng/mL)	Pure found^b^ (ng/mL)	Pure recovery (%*R*)
**Dapagliflozin**	300	301.11	100	98.51	98.51
			300	297.54	99.18
			400	402.16	100.54
			500	504.85	100.97
	Mean ± %RSD				99.80 ± 1.152
**Sitagliptin**	600	599.70	200	203.55	101.78
			400	407.37	101.84
			800	796.16	99.52
			1000	1007.40	100.74
	Mean ± %RSD				100.97 ± 1.080

^a^ Average of five determinations.

^b^ Average of three determinations.

### Pharmaceutical applications

The proposed method was successfully applied to the analysis of dapagliflozin and sitagliptin in their commercial pharmaceutical formulations. The obtained results were in good agreement with the labeled amounts, indicating the absence of interference from excipients. Statistical comparison with the reported methods using Student’s t-test and F-test revealed no significant difference at the 95% confidence level, confirming the accuracy and precision of the proposed method.

The comparison with the reported spectrophotometric methods was performed only for pharmaceutical dosage form analysis and was not intended to evaluate plasma applications. Although the comparison was performed against previously reported spectrophotometric methods, the proposed method offers the additional advantage of simultaneous determination of both analytes without chromatographic separation.

The statistical comparison is summarized in Table 4.

**Table 4:** Determination of dapagliflozin and sitagliptin in pharmaceutical preparations by the proposed first derivative synchronous spectrofluorimetric and reported methods:.

Parameters	Proposed method		Reported methods ^(14,15)^	
	Dapagliflozin	Sitagliptin	Dapagliflozin	Sitagliptin
Number of measurements	5	5	5	5
Mean % recovery	100.37	99.95	99.38	100.94
% RSD	0.805	0.882	1.119	0.768
Variance	0.652	0.601	1.236	0.778
Student’s *t*-test*	1.611 (2.306)	1.874 (2.306)	——	——
*F*-value*	1.896 (6.388)	1.294 (6.388)	——	——

* The values in parenthesis are tabulated values of “*t* ” and “*F* ” at (P = 0.05).

### Spiked human plasma application

The applicability of the proposed method to biological samples was evaluated using spiked human plasma. The spiked plasma samples were analyzed within the same concentration ranges established in the calibration study. The method demonstrated satisfactory recoveries, indicating its suitability for the determination of both drugs in biological matrices. Slightly lower recovery values compared to pharmaceutical formulations may be attributed to matrix effects and partial loss during protein precipitation and extraction steps. These results demonstrate the feasibility of the proposed method for the determination of dapagliflozin and sitagliptin in spiked human plasma samples.

The obtained results are presented in Table 5.

**Table 5:** Determination of dapagliflozin and sitagliptin in spiked human plasma by the proposed method:.

Dapagliflozin			Sitagliptin		
Added (ng/mL)	Found* (ng/mL)	%Recovery	Added (ng/mL)	Found* (ng/mL)	%Recovery
50	46.94	93.88	1800	1723.54	95.75
100	93.82	93.82	1200	1167.31	97.28
300	281.68	93.89	800	765.87	95.73
600	571.92	95.32	400	384.06	96.02
800	747.75	93.47	100	92.91	92.91
Mean ± %RSD		94.08 ± 0.762	Mean ± %RSD		95.54 ± 1.676

* Average of six determinations.

## Conclusion

A novel, sensitive, simple, and cost-effective first-derivative synchronous spectrofluorimetric method was successfully developed and validated for the simultaneous determination of dapagliflozin and sitagliptin. To the best of our knowledge, this is the first validated first-derivative synchronous spectrofluorimetric method reported for the simultaneous determination of both drugs without chromatographic separation. The proposed method effectively resolved the severe spectral overlap between both drugs without the need for prior separation, providing a simple and rapid analytical alternative. The method demonstrated excellent linearity within concentration ranges covering the reported maximum plasma concentrations (Cmax), along with high accuracy, precision, and robustness in accordance with ICH validation guidelines. In addition, the method showed satisfactory applicability for the analysis of pharmaceutical formulations and spiked human plasma samples. Compared with conventional chromatographic techniques, the proposed approach offers significant advantages in terms of simplicity, cost-effectiveness, reduced analysis time, and minimal solvent consumption, making it well-suited for routine quality control and the analysis of spiked human plasma samples. Overall, the developed method represents the first validated first-derivative synchronous spectrofluorimetric approach for the simultaneous determination of dapagliflozin and sitagliptin, providing a practical analytical alternative for pharmaceutical analysis and spiked human plasma samples.

## Data Availability

The datasets generated and/or analysed during the current study are included in the published article. Any additional data supporting the findings of this study are available from the corresponding author upon reasonable request.
